# Failed Stabilization for Long-Term Potentiation in the Auditory Cortex of *Fmr1* Knockout Mice

**DOI:** 10.1371/journal.pone.0104691

**Published:** 2014-08-12

**Authors:** Sungchil Yang, Sunggu Yang, Jae-Sung Park, Alfredo Kirkwood, Shaowen Bao

**Affiliations:** 1 Helen Wills Neuroscience Institute, University of California, Berkeley, California, United States of America; 2 Department of Neuroscience, Johns Hopkins University, Baltimore, Maryland, United States of America; University of Southern California, United States of America

## Abstract

Fragile X syndrome is a developmental disorder that affects sensory systems. A null mutation of the Fragile X Mental Retardation protein 1 (*Fmr1*) gene in mice has varied effects on developmental plasticity in different sensory systems, including normal barrel cortical plasticity, altered ocular dominance plasticity and grossly impaired auditory frequency map plasticity. The mutation also has different effects on long-term synaptic plasticity in somatosensory and visual cortical neurons, providing insights on how it may differentially affect the sensory systems. Here we present evidence that long-term potentiation (LTP) is impaired in the developing auditory cortex of the *Fmr1* knockout (KO) mice. This impairment of synaptic plasticity is consistent with impaired frequency map plasticity in the *Fmr1* KO mouse. Together, these results suggest a potential role of LTP in sensory map plasticity during early sensory development.

## Introduction

Fragile X syndrome (FXS) is the most common cause of heritable mental retardation. Expansion of trinucleotide CGG repeats in the *Fmr1* gene results in hypermethylation and loss-of-function of the gene [1]. *Fmr1* encodes Fragile X mental retardation protein (FMRP), an mRNA-binding protein that regulates local mRNA translation. FMRP is expressed in layer IV of sensory cortex, with peak expression in the critical period of cortical plasticity, during which pronounced synaptogenesis and dendritic rearrangement occur, suggesting its role in developmental plasticity [2], [3]. Lack of FMRP exaggerates mGluR-stimulated protein synthesis and has been hypothesized to underlie many of the abnormal phenotypes of FXS [4], [5].

Maladaptive sensory responses and impaired sensory integration are characteristic of FXS patients and model animals [6]–[9]. Studies of the *Fmr1* KO mouse indicate that the null mutation has different effects on development in visual and somatosensory systems. For example, developmental sensory map plasticity is altered in the visual cortex [10], but appears to be relatively normal in the barrel cortex [2]. Deletion of the *Fmr1* gene also differentially influences synaptic plasticity in different sensory systems. For example, while LTP is impaired in the visual cortex of *Fmr1* KO [11], it is delayed in barrel cortex of *Fmr1* KO mice, even though barrel map plasticity appeared normal [2]. Therefore, it is still unclear how altered synaptic plasticity could impact experience-dependent development of the sensory systems in the Fragile X model animals.

Fragile X syndrome has an auditory component, which is manifested as altered sensitivity to sounds, impaired integration of acoustic stimuli, and delayed language development, suggesting that development and plasticity in the auditory system may also be compromised [6]–[9]. A recent study revealed that, unlike in the somatosensory and visual systems where sensory map plasticity is either normal or present in an altered form, experience-dependent reorganization of the sound frequency map is grossly impaired in the *Fmr1* KO mice [12]. This feature provides a powerful model to investigate synaptic mechanisms underlying the impaired sensory development and cortical functions in Fragile X syndrome.

We examined LTP in *Fmr1* KO mice, and found that LTP could be readily induced in auditory cortex of wildtype mice during a period of postnatal 2^nd^-3^rd^ weeks, but was dramatically reduced after the 4^th^ week. Furthermore, *Fmr1* KO mice also lacked LTP in the auditory cortex both during postnatal 2^nd^-3^rd^ weeks and after the 4^th^ week. These results suggest the possibility that the deficient auditory cortical LTP might contribute to the frequency map plasticity impairment observed in the *Fmr1* KO mouse.

## Experimental Procedures

### Animals

All procedures used in this study were approved by the Animal Care and Use Committee at the University of California, Berkeley. Wild-type (WT) and *Fmr1* knock-out (KO) mice on the FVB background were originally obtained from The Jackson Laboratory (FVB.129P2-Pde6b^+^ Tyr^c-ch^/AntJ and FVB.129P2-Pde6b^+^ Tyr^c-ch^ Fmr1^tm1Cgr^/J). Only male homozygous WT or KO mice were used in this study.

### Brain slice preparation for physiology

Primary auditory cortical slices were prepared from mice in two developmental groups: P16-P20 (early-age group) and P27-P31 (late-age group). Animals were deeply anesthetized with isoflurane. The brains were quickly removed and placed into chilled (4°C), oxygenated (5% CO_2_ and 95% O_2_) slicing medium containing (in mM): 212.7 sucrose, 5 KCl, 1.23 NaH_2_PO_4_, 26.0 NaHCO_3_, 11.0 glucose, 1.5 MgCl_2_, 2.5 CaCl_2_. Coronal auditory slices (400 µm) were prepared with a vibratome. Slices were then transferred to a holding chamber containing oxygenated physiological saline made up of (in mM): 124.0 NaCl, 4 KCl, 1.23 NaH_2_PO_4_, 26.0 NaHCO_3_, 10.0 glucose, 1.5 MgCl_2_, 2.5 CaCl_2_. After at least 1 hr of recovery, individual slices were transferred to a submersion-type recording chamber and oxygenated physiological saline was continuously superfused at a rate of 2 ml/min at 32°C temperature.

### Extracellular field Recording

**Figure 1:**
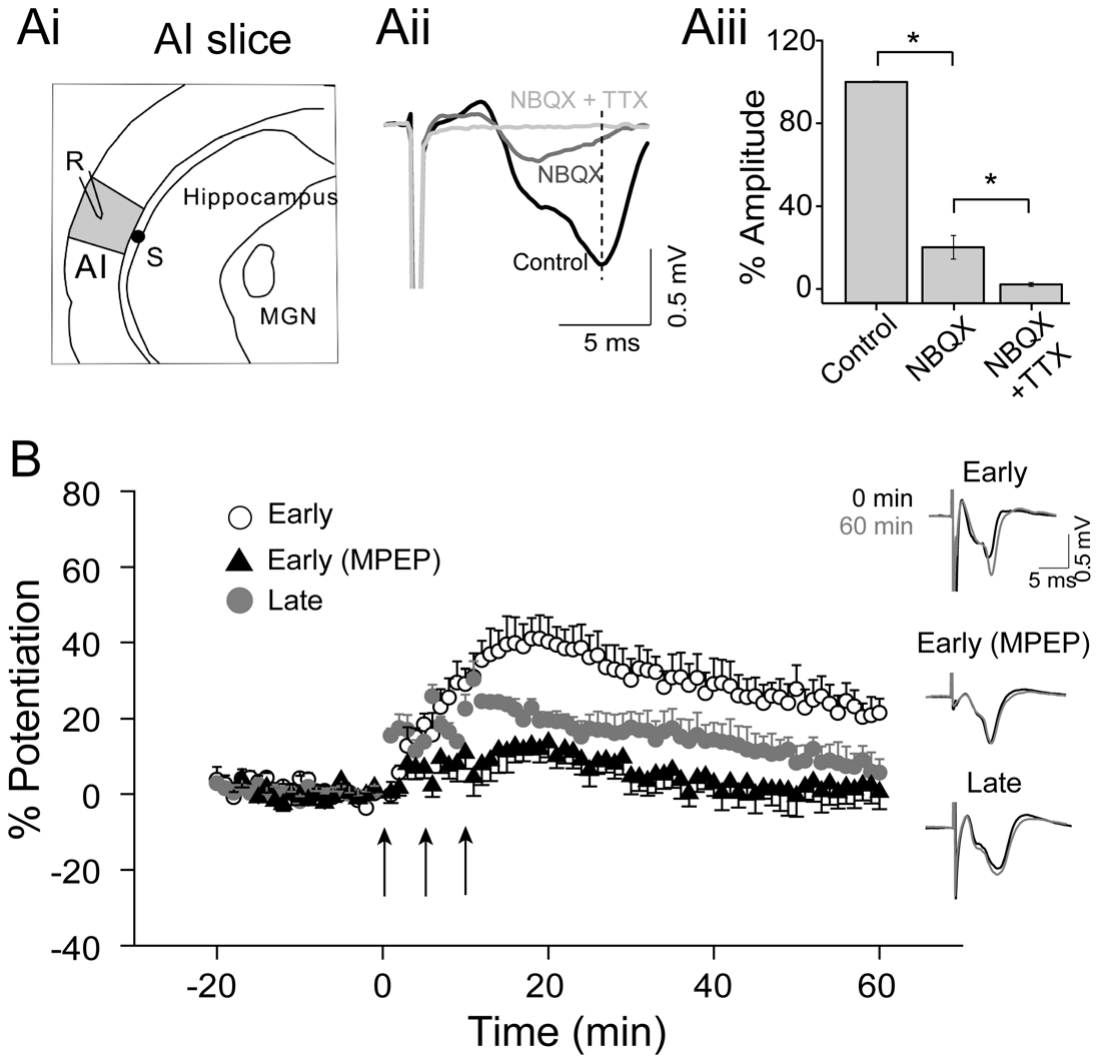
Differential LTP expression in early- (P16-20) and late-age (P27-31) windows. **Ai**. A schematic of experimental setup. S, stimulus (black dot); R, recording (Layer III/IV). **Aii**. Field potential responses in the presence of NBQX and/or TTX. **Aiii**. Field potential amplitude measured at the peak latency (see dashed vertical line in **Aii**) was reduced by 80% by NBQX, and was completely abolished by further TTX application. **B**. Average responses in early- and late-age groups. Note the significant LTP in the early-age group but not in the late-age group. LTP in the early-age group was completely blocked by MPEP application. Insets show overlapping field potential traces before (black) and 60 min after (gray) LTP induction. Arrows indicate three consecutive tetanic stimulations. *, p<0.05.

Extracellular field potential recording procedure had been previously reported [13], [14]. Extracellular field potentials were recorded in the absence of GABA receptor antagonists, using glass pipettes filled with ACSF (5–10 MΩ). Synaptic responses were evoked through a concentric bipolar stimulating electrode (FHC, 100 µm o.d.). When a single electric microstimulation was applied to the underlying white matter, field potentials recorded from layers III/IV of the primary auditory cortex showed the characteristic negative waveform (Figure 1, Ai). Bath application of 10 µM NBQX (Abcam) reduced the field potential amplitude by 80% (Figure 1, Aii-Aiii; NBQX = 20.09±5.70% measured at the peak of the field potential; n = 4, *P*<0.01, paired-*t* test), indicating that the field potential amplitude was largely determined by AMPA-mediated responses. Application of 1 µM TTX (Tocirs) nearly completely abolished the remaining field potential (2.07±0.93%; n = 4, *P*<0.01 compared to NBQX alone, paired-*t* test). Since the non-AMPA component of the field potential peaked early compared to the full field potential, it could potentially contaminate the measurement of the field potential slope. We therefore used the peak, but not the slope, of the field potential for our LTP measurement. Field potential amplitude had been used in earlier studies of cortical LTP [11], [13], [15]. The amplitude of the field potential was measured. After stable postsynaptic responses were maintained for 20 minutes, three tetanic stimulations (100 Hz, 1-s duration, 5-min intervals) were applied. Baseline responses were recorded using half-maximal stimulation intensity at 0.033 Hz. LTP was induced by three repetitions of 100-Hz stimulation of 1-s duration.

All data are presented as mean ± SEM. One-sample t-test was used to determine the statistical significance of PTP and LTP. ANOVA was used to compare LTP magnitudes between experimental groups. The LTP decay rate was calculated for individual experiments and averaged for each group.

## Results

### mGluR-dependent LTP in the auditory cortex

Three repetitions of tetanic stimulation resulted in long-lasting LTP in the early-age group (P16-20, WT early-age group at 60 min, n = 6, 122.96±4.46%; *p* = 0.0036, compared to baseline with one-sample t-test; see Figure 1B). Previous studies have shown that LTP in the visual and auditory cortex is mGluR5-dependent [11], [16]. In the present study, bath-application of 50 µM MPEP, an mGluR5 inhibitor, completely blocked LTP in the auditory cortex measured at 60 min post-induction (MPEP-treated WT early-age group, n = 6, 101.14±5.85%, *p*>0.5, compared to baseline; see Figure 1B), indicating that the LTP in auditory cortex layer III/IV is also mGluR5-dependent.

### Age-dependent LTP in the auditory cortex

**Figure 2:**
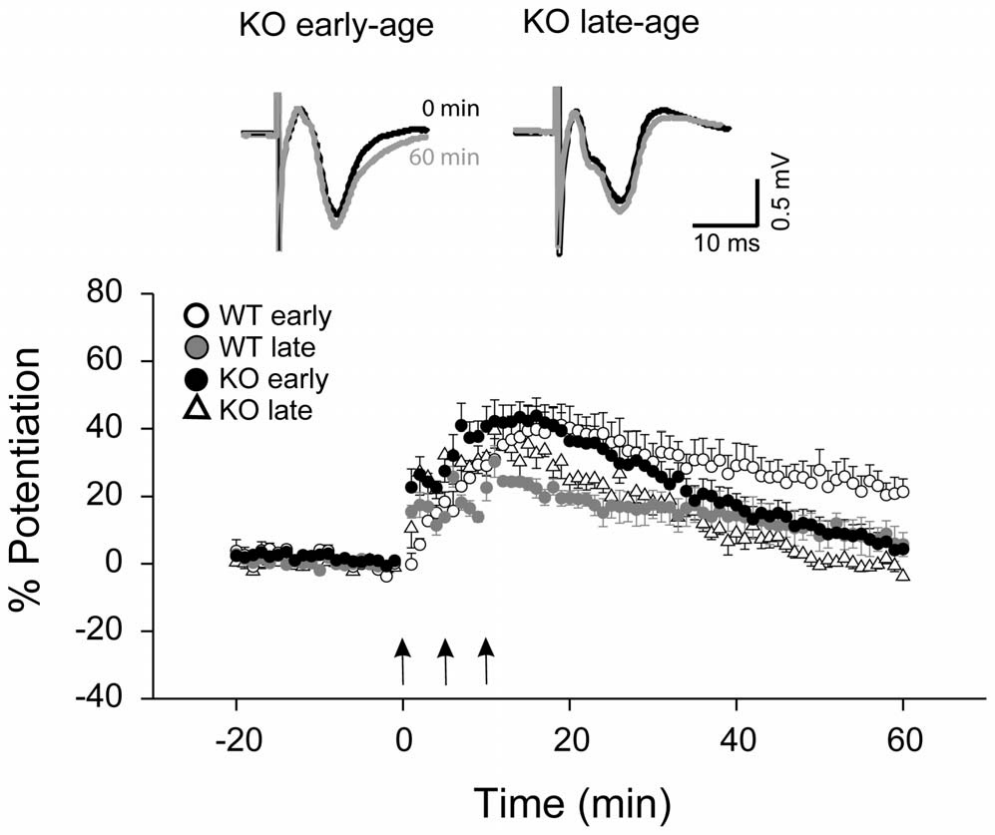
Lack of LTP expression in Fmr1 KO in both early- and late-age windows. Average responses over time are shown for two different age windows (early-age: P16-20, n = 6; late-age: P27-31, n = 4). Insets show overlapping field potential traces before (black) and 60 min after (gray) LTP induction. Note that there was no significant LTP in early- and late-age groups of *Fmr1* KO mice. Arrows indicate three consecutive tetanic stimulations.

**Figure 3:**
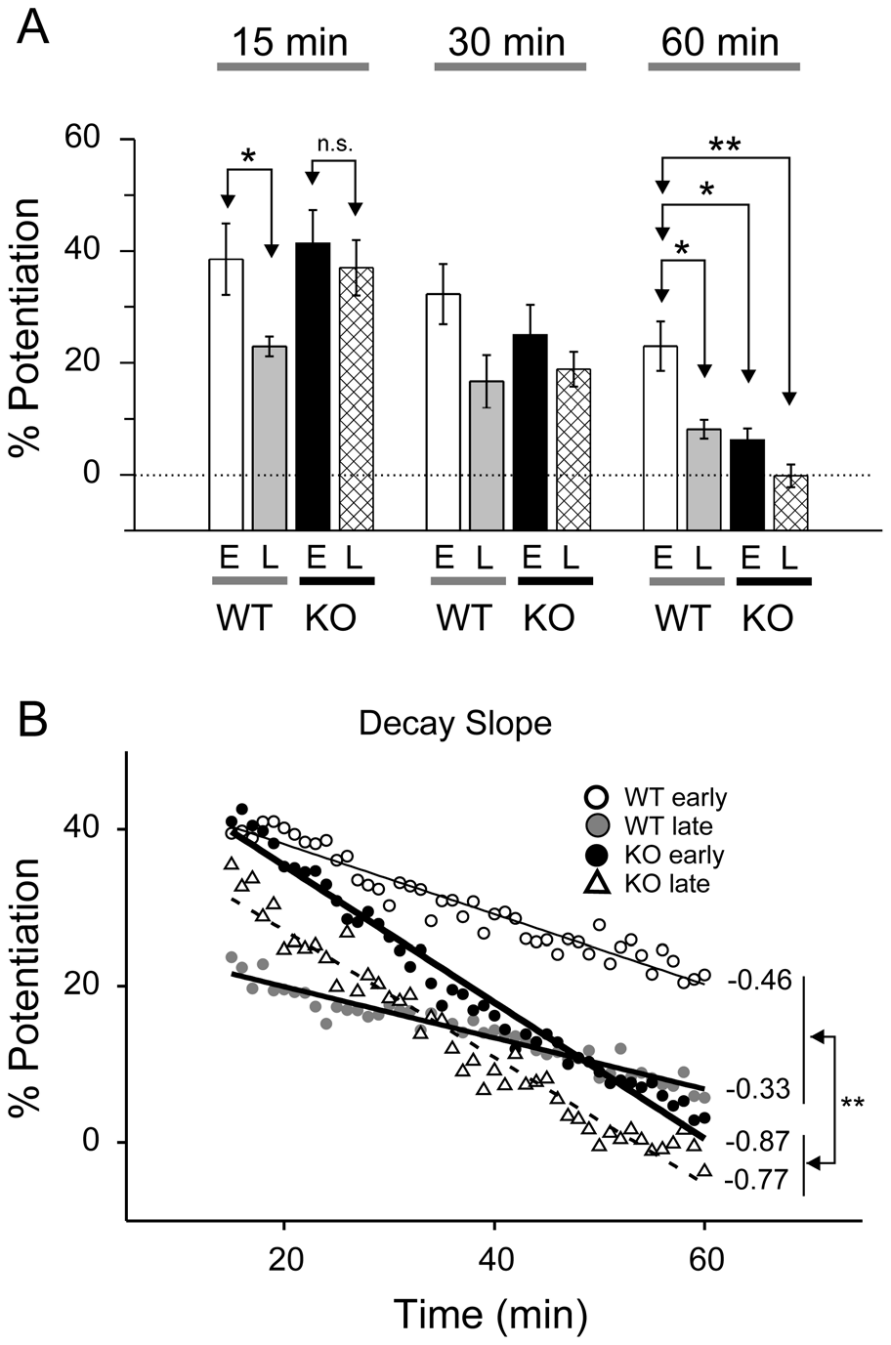
Destabilization of LTP in Fmr1 KO mice. **A**. Average synaptic potentiation shown in different time windows (15, 30 and 60 min). **B**. Decay slope in four groups (WT early, WT late, KO early and KO late). Note the steeper decay in early and late window of Fmr1 KO mice compared to those in early and late window of Fmr1 WT mice. *, *p*<0.05; **, *p*<0.01; n.s, not significant.

Thalamocortical synapses in somatosensory and visual cortex exhibit LTP only in early stages of respective cortical development [17], [18]. We examined whether LTP in the auditory cortex was also restricted to an early age of development. Tetanic stimulations produced substantial post-tetanic potentiation (PTP) (n = 6, 138.54±6.40%, measured at 15 min after tetanic stimulation; *p* = 0.0036, compared to baseline; Figures 1B, 2 and 3) and LTP (see above) in the early-age group (P16-20). In contrast, the PTP was reduced by 40% in the late-age group compared to early-age group (P27-31; 122.90±1.76%, n = 4), and decayed steadily to the baseline level within 60 min (LTP, 108.15±4.18%, n = 4; *p* = 0.147, compared to baseline). A age-by-decay time 2-way ANOVA revealed significant effects of age (*F*_24,1_ = 13.72, *p* = 0.0011) and decay time (15, 30 and 60 min: *F*_24,2_ = 3.85, *p* = 0.035; see Figure 3), but no effect of their interaction (*F*_24,2_ = 0.025, *p* = 0.98). These results suggest that the late-age group had significantly reduced PTP and LTP compared to the early-age group, but their post-tetanus decay rates were not significantly different.

### Destabilization of LTP in Fmr1 KO mice

LTP is impaired in the visual cortex, and is delayed in the somatosensory cortex of Fmr1 KO mice [2], [11]. We examined PTP and LTP in auditory cortex of *Fmr1* KO mice in the early- and late-age groups. We observed PTP of the postsynaptic response following the tetanic stimulation in both early- and late-age groups (Figure 2 and 3A). Both the early- and late-age KO groups showed significant PTP (KO early-age group: 141.39±5.94%, n = 9; KO late-age group: 137.00±4.97%, n = 5; *p*<0.01 for both groups compared to baseline), but potentiation rapidly decayed back to the baseline levels within 60 min (KO early-age group: 106.25±4.49%, n = 9; KO late-age group: 101.06±1.33%, n = 4; *p*>0.2 for both KO groups, compared to baseline). A age-by-decay 2-way ANOVA showed a significant effect of decay time (*F*_35,2_ = 19.32, *p*<0.0001), but no effect of age (*F*_35,1_ = 1.29, *p* = 0.26) or age-by-decay time interaction (*F*_35,2_ = 0.013, *p* = 0.99). These results indicate that PTP and LTP in the KO mice did not show age-dependence.

To compare between WT and KO mice, we performed a genotype-by-age-by-decay time 3-way ANOVA. We found a genotype x decay time interaction (*F*_59,2_ = 3.53, *p* = 0.036), but no main effect of genotype (*F*_59,1_ = 0.291, *p* = 0.59) or genotype x age interaction (*F*_59,1_ = 2.685, *p* = 0.11). These results indicate that WT and KO mice showed different field potential decay rates after tetanic stimulation.

We calculated the decay rate as the slope of the linear regression of the field potential amplitude 15–60 min after the tetanic stimulation. The decay rate was similar between early- and late-age groups but was steeper for *Fmr1* KO than WT group (Figure 3B decay slope; KO early-age group: −0.87±0.13, n = 9; KO late-age group: −0.77±0.08, n = 4; WT early-age group: -0.46±0.11, n = 6; WT late-age group: −0.33±0.08, n = 4; genotype-by-age 2-way ANOVA, genotype effect, *F*_19,1_ = 10.34, *p* = 0.0045; age effect, *F*_19,1_ = 0.80, *p* = 0.38; interaction, *F*_19,1_ = 0.017, *p* = 0.90).

The similar PTP amplitudes and decay rates and the lack of LTP in both *Fmr1* KO early- and late-age groups suggest that the age dependence of PTP and LTP observed in WT mice is not present in the *Fmr1* KO mice. The faster decay of the tetanic stimulation-induced potentiation in the *Fmr1* KO mice suggests that LTP is unstable in the *Fmr1* KO mice.

## Discussion

**Figure 4:**
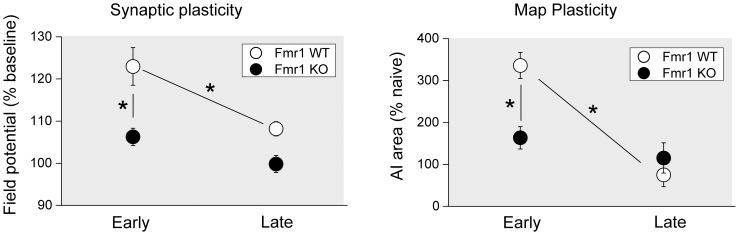
Comparison of synaptic and map plasticity at different ages. Both synaptic (i.e., LTP) and map plasticity (i.e., map expansion following tone exposure) are observed in early window, and only in WT mice but not in *Fmr1* KO mice. Map plasticity data adapted from a previous report [12]. Error bars represent SEM. *, *p*<0.05.

Multiple neural mechanisms are hypothesized to determine the critical period window of developmental plasticity in the visual, somatosensory and auditory cortices [19]–[25], including the expression of LTP and LTD [14], [17], [18], [23]. The present study indicates that, a long-lasting (≥60 min) form of LTP was expressed in P16-21 but not P27-31 animals. Long-lasting LTP has also been reported in layer VI pyramidal neurons in gerbil auditory cortex between P14 and P21 [26]. Previous reports have demonstrated LTP in layers II/III auditory cortex of 4–7 weeks old rats [27], [28]. However, since LTP in those studies was only monitored for 30–40 min, it is unclear whether long-lasting LTP was induced. Our findings are consistent with early reports of distinct critical periods for LTP in the visual and somatosensory cortices. The critical periods for LTP in visual and barrel cortex coincide with the critical periods for ocular dominance plasticity and barrel map plasticity [14], [17], [18], [23]. In the auditory cortex, the critical period for frequency map plasticity is reported to be P11 to P13 for Sprague-Daley rats and P11 to P15 for C57B/6 mice [22], [29]. However, other aspects of sensory representation can still be altered by experience after closure of the frequency map critical period [30], [31]. For example, exposure to a frequency-modulated tone can alter tuning bandwidth of cortical neurons in a period from P16 to P23 and change frequency modulation selectivity from P24 to P39 [32]. In addition, we have also observed strain differences in the critical period of frequency map plasticity—the critical period occurred in some strains of mice in a later period from P16 to P23 (Hamilton and Bao, unpublished observation). Thus, the observed LTP in WT mice from P16 to P20 might be a correlate of the tuning bandwidth plasticity. It is also possible that the critical period for frequency map plasticity is delayed in this strain of mice, in which case the LTP we observed could be a correlate of map plasticity (Figure 4).

Unlike in the WT mice, which showed more PTP and LTP in the early- than late-age group, the *Fmr1* KO mice exhibited similar levels of PTP, but lacked LTP in both groups. In both early- and late-age groups, the induced potentiation decayed more rapidly in *Fmr1* KO mice than in WT mice. The late-age group KO mice showed higher potentiation compared to late-age group WT mice at 15 min after the initial LTP induction. The potentiated response in the late-age group KO mice decayed rapidly to the baseline level by 60 min after LTP induction. These results suggest that *Fmr1* KO mice have a prolonged window for PTP, but potentiation is unstable and decays more rapidly. Although these results are different from the impaired LTP and enhanced LTD in the visual cortex, or the delayed critical period for LTP in the somatosensory cortex, they are consistent with how disrupted protein synthesis in *Fmr1* KO mice may affect the LTP. Late-phase LTP requires protein synthesis [33], [34]. In the presence of protein synthesis inhibitor, LTP induction protocol leads to synaptic potentiation, but the potentiated synaptic responses decay gradually [33], [34], similar to the destabilized LTP that we observed in the auditory cortex of *Fmr1* KO mice. One of the consequences of *Fmr1* null mutation is dis-regulated local protein synthesis [4], [5], [35], which could conceivably disrupt late-phase LTP.

Potentiation of thalamocortical synapses has been shown to reduce the detection threshold and sharpen the tuning of neurons in the auditory cortex [36]. Adaptive auditory map plasticity shapes perception and perceptual sensitivity [37], [38]. Thus, the unstable cortical LTP and the resulting lack of sensory map plasticity could contribute to the impaired sensory functions and speech development in Fragile X syndrome [7]–[9], [39], [40].

Cortical sound representations, including frequency map, tuning bandwidth, and response thresholds, develop normally in *Fmr1* KO mice [12]. However, experience-dependent frequency map reorganization is severely impaired in the *Fmr1* KO mice [12]. Exposing WT mice to 16-kHz tone pips in an early (P9-20) but not late window (P20-30) enlarged the cortical area tuned to that frequency. However, exposure in neither window was able to alter the frequency map in *Fmr1* KO mice. The parallel between impaired LTP and frequency map plasticity suggests the intriguing possibility that they are causally correlated (Figure 4). The failed stabilization of LTP in the *Fmr1* KO mice observed in the present study further suggests that changes to the sensory map are not consolidated into long-lasting memory traces. Enhancing memory consolidation may thus improve learning in Fragile X patients.
